# Case Study on Promoting Strategies and Coping with Difficulties in Excellent Intergenerational Programs

**DOI:** 10.1093/geroni/igaf122.3158

**Published:** 2025-12-31

**Authors:** Stephanie Yu-Ching Chen

**Affiliations:** National Chung Cheng University, Minxiong, Chiayi, Taiwan (Republic of China)

## Abstract

Eleven units that promote excellent intergenerational programs were selected for the case study to improve the quality of intergenerational programs and effectively construct a training model for intergenerational professionals through the recommendation of the Ministry of Education and the Ministry of Health and Welfare in Taiwan. The research subjects include five organizational types: senior learning centers hosted by primary schools, senior learning centers hosted by community organizations, universities that promote intergenerational learning programs, community care centers, and elderly day care centers. 19 relevant personnel were interviewed, and relevant documents and information were collected. The research results found that (1) the purpose of promoting intergenerational programs is that schools aim to cooperate with the government in promoting senior education policies and reducing age discrimination among students, while community organizations aim to reduce generational gaps and social isolation and increase community inclusiveness. But whether it is a community or a school, they all hope to enhance social cohesion through interaction between different generations. (2) Difficulties in promotion and coping strategies: Difficulties on the school side include low willingness of teachers to participate, insufficient manpower to organize the program, difficulty in balancing the needs of different generations in curriculum design, and parents’ concerns about the safety of the elderly entering campus. The difficulties faced by the community are mainly insufficient funding and manpower, generational barriers, and diverse community needs. Schools and communities have different response strategies depending on organizational attributes and resources. Finally, we propose strategies to promote intergenerational programs.

